# The Effects of Scleral Collagen Cross-Linking Using Glyceraldehyde on the Progression of Form-Deprived Myopia in Guinea Pigs

**DOI:** 10.1155/2016/3526153

**Published:** 2016-07-18

**Authors:** Yanhua Chu, Zhaohui Cheng, Jing Liu, Ying Wang, Haixia Guo, Quanhong Han

**Affiliations:** ^1^Tianjin Eye Hospital, Clinical College of Ophthalmology Tianjin Medical University, No. 4 Gansu Road, Heping District, Tianjin 300020, China; ^2^Tianjin Medical University Eye Hospital, Tianjin Medical University Eye Institute, No. 251 Fukang Road, Nankai District, Tianjin 300384, China

## Abstract

To investigate the effects of collagen cross-linking using glyceraldehyde on the biomechanical properties of the sclera and the axial elongation of form-deprived myopia in the guinea pig. Thirty-six guinea pigs were randomly assigned to four groups: FDM (form-deprived myopia); FDMG (form-deprived myopia treated with glyceraldehyde); FDMS (form-deprived myopia treated with 0.9% isotonic sodium chloride); and normal control (free of form-deprivation). FDM was achieved in the right eye using a latex facemask. The right eye in FDMG was treated with a posterior subtenon injection of 0.5 M glyceraldehyde; 0.9% isotonic sodium chloride was administered to the right eye in FDMS group using the same method. Axial length, refraction, and stress-strain of the sclera were measured at scheduled time points. The treated eyes were also examined histologically by light microscopy. It was found that glyceraldehyde treatment significantly increased the stiffness of the sclera in the FDM eyes and abnormalities have not been observed in the retina and optic nerve of the treated eyes. But the development of myopia was not affected.

## 1. Introduction

Progressive myopia is an important unsolved problem in ophthalmology. There has been an overall increase in the prevalence of myopia worldwide. The incidence of myopia in the USA and Europe is reported to be around 30%, and in Asian countries it affects around 60% of the general population [1, 2]. Pathologic myopia of more than −6.0 D can be found in 12–15% of all myopic patients [2]. Low to moderate degrees of myopia can be easily corrected using optical or refractive surgical means. Pathologic myopia, while also correctable using these optical approaches, is of major concern because of sight-threatening consequences such as retinal detachment, macular schisis, and macular degeneration [3]. All these complications are associated with progressive axial elongation which cannot be treated by refractive means.

Treatments on retarding axial elongation in myopia remain limited and controversial. The pharmacological treatment currently in use is topical atropine, which is accompanied by numerous side effects and offers little benefit for already highly myopic eyes [4, 5]. Surgical approaches include scleral reinforcement surgery in which donor sclera or synthetic bands are placed around the back of the globe and sutured to the sclera to provide scleral support or injection of a polymeric composition forming a gel under Tenon's capsule and inducing scar tissue to prevent axial elongation [6–8]. However, the outcomes of all of these surgical therapies are controversial and surgical trauma also should be taken into consideration.

There is strong evidence from clinical and experimental studies indicating that the biochemical and biomechanical properties of the sclera play a major role in the progression of myopia [9]. Thinning of the sclera and weakened biomechanical properties, particularly at the posterior pole of the eye, have long been known to be an important feature in the development of high myopia in human and mammalian models [2]. Thus, the sclera has been considered to be a prime target for therapeutic manipulation regarding myopia progression. As mentioned above surgical treatments based on sclera reinforcement have been evaluated. Because of the controversial results and complications, the various surgical approaches have not been widely applied in clinical practice.

Animal studies involving tree shrews have shown that impaired collagen cross-linking is an important factor in the weakening process of the myopic sclera [10]. Indeed, collagen cross-linking has been successful in treating progressive keratoconus in which the cornea undergoes a thinning process and exhibits weakened biomechanical properties, as in the myopic sclera [11, 12]. Several studies have attempted to increase the rigidity of the sclera through collagen cross-linking. Collagen cross-linking induced by the photosensitizer riboflavin and ultraviolet A (UVA) has been shown to lead to a significant increase in Young's modulus for treated porcine, rabbit, and human sclera [13, 14]. However, scleral cross-linking using riboflavin and UVA requires an operation entailing surgical exposure of the posterior sclera and has a potential cytotoxic risk for the retina [14].

An alternative method of chemical cross-linking using glyceraldehyde has been shown to significantly increase Young modulus in porcine sclera* in vivo* and in rabbit sclera* in vitro* [13, 15]. Another study involving the rabbit demonstrated that the efficacy of glyceraldehyde in increasing scleral biomechanical strength can extend over a period of ≤8 months [16]. Glyceraldehyde is used for tissue engineering in the pharmaceutical and food industry and is generally considered nontoxic [15, 17]. The safety of glyceraldehyde treatment on eyes has also been studied by Wollensak and Iomdina [18]. Light microscopy examination revealed that there were no abnormalities in the optic nerve and retina, and only some moderate infiltration of neutrophils and bleeding adjacent to the injection site were found in several animals [18]. Unlike riboflavin cross-linking and sclera reinforcement surgery, glyceraldehyde can be easily administered by means of sequential parabulbar injections and have large treatment area. Consequently, scleral cross-linking with glyceraldehyde may be a promising method for the treatment of myopia. Although glyceraldehydes could improve sclera rigidity in normal animal eyes, its effect on progressive myopic eyes has not yet been studied.

The aim of the present study was to investigate the effectiveness of collagen cross-linking using glyceraldehyde in increasing scleral rigidity and retarding the axial elongation in form-deprived myopia (FDM) of guinea pig.

## 2. Materials and Methods

This study was approved by the Animal Care and Ethics Committee at Tianjin Medical University (Tianjin, China). The treatment and care of animals were conducted according to the ARVO Statement for the Use of Animals in Ophthalmic and Vision Research.

Thirty-six pigmented guinea pigs (*Cavia porcellus*; approximately 3 weeks old) were obtained from the Animal Breeding Unit at Tianjin Medical College and randomly assigned to the following four groups with nine animals per group: FDM (form-deprived myopia); FDMG (form-deprived myopia treated with glyceraldehyde); FDMS (form-deprived myopia treated with 0.9% isotonic sodium chloride); and normal control (free of form deprivation). All animals underwent biometric measurement (refraction and axial length) prior to the experiment. FDM was achieved by means of a latex facemask covering the right eye. The right eye in the FDMG group was treated using a sub-Tenon injection of 0.5 M glyceraldehyde; 0.9% isotonic sodium chloride was administered to the right eye in the FDMS group using the same method. Biometric measurement was undertaken at four time points (0, 2, and 4 weeks). Following the final ocular measurements, the right eyes were enucleated after the animal had received an overdose of anesthesia. The stress-strain of the sclera was measured and histological examination was undertaken with light microscopy.

### 2.1. Form Deprivation

**Figure 1:**
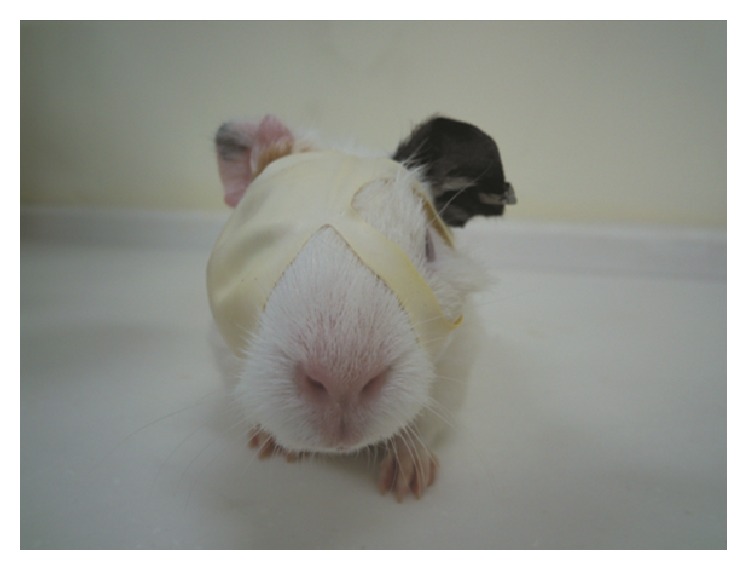
Form-deprived myopia in guinea pig achieved by latex facemask.

FDM was achieved using a latex facemask (OuJie, Suzhou, China) covering the right eye. The left eye, nose, and both ears remained exposed, as described by Lu et al. [19] (Figure 1). The facemasks were examined once daily to ensure that they were in place and fitted well.

### 2.2. Treatment Procedures

After topical anesthesia using 0.5% proparacaine hydrochloride (Alcon, Purrs, Belgium), sub-Tenon injections of 0.05 mL of 0.5 M glyceraldehydes (Sigma-Aldrich, Steinheim, Germany) dissolved in physiologic saline solution were administered as a depot with the injection site 3.0 mm behind the limbus in the superonasal and inferotemporal quadrant, respectively; then the injection site was transferred to the other two quadrants for every other injection; a 1.0 mL tuberculin syringe with a sharp 25-gauge injection needle was used. The first injection was given at day 1 just before achieving FDM and the injections were given twice a week. 0.05 mL of 0.9% isotonic sodium chloride solution (Dazhong, Tianjin, China) was administered in the same way. Ofloxacin eyedrops (Santen, Osaka, Japan) were applied four times a day [20].

### 2.3. Biometric Measurement

#### 2.3.1. Retinoscopy

Retinoscopy for all animals was performed by the same optometrist in a dark room using a streak retinoscope. Before examination, 1% cyclopentolate hydrochloride (Alcon) was topically administered to the eye every 5 min for four repetitions to achieve a completely dilated pupil. The refraction was recorded as the mean value of the horizontal and vertical meridian [19, 21, 22].

#### 2.3.2. Ultrasonography

After topical anesthesia with 0.5% proparacaine hydrochloride (Alcon), ocular axial length was measured using an A-scan ultrasonography (Cinescan A/B, Quantel Medical, Clermont Ferrand, France). The ultrasound frequency was 11 MHz. Sound velocities were assumed to be 1557.5 m/s for the anterior segment, 1723.3 m/s for the lens, and 1540 m/s for the vitreous humor [19, 23, 24]. The ultrasound data represented the mean of 10 repeated measurements.

### 2.4. Stress-Strain Measurements

#### 2.4.1. Specimen Preparation

The animals were sacrificed by overdose anesthesia with 160 mg/kg Pentobarbital Sodium (Sigma-Aldrich, Steinheim, Germany). After making a complete circular incision located at a distance 1 mm anterior the limbus, the posterior eye cup was turned around using a cotton tip applicator. The retina and choroid were removed. Two scleral strips of width 2 mm were dissected sagittally using a double-shade shaver, from the nasal and temporal margin of the optic nerve to the anterior end almost at 1 and 11 o'clock. Care was taken to insure that all strips were cut in a similar orientation to minimize any differences resulting from possible anisotropy. The thickness of the scleral strips was determined using a mechanical micrometer caliper [24].

#### 2.4.2. Stress-Strain Test

**Figure 2:**
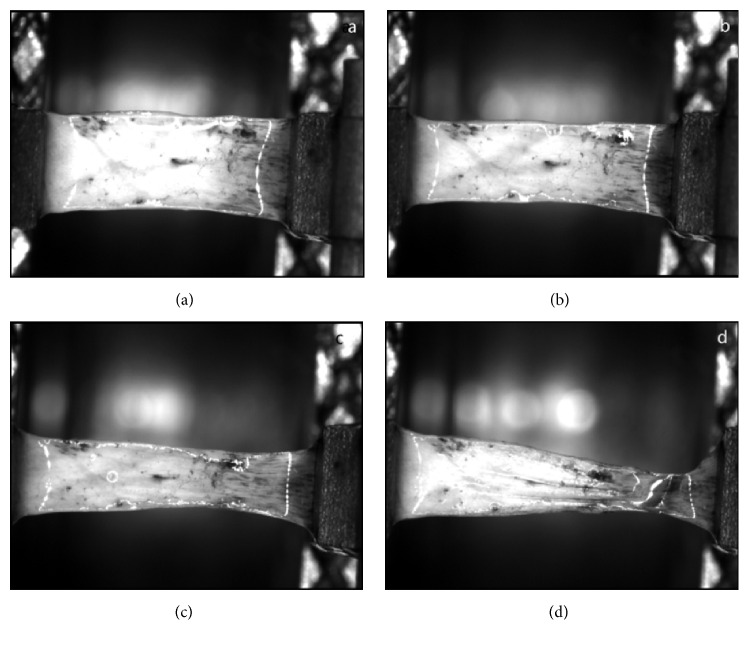
Stress-strain measurement of the scleral strip. ((a)–(d)) Showing that together with increased stress, the strain in the sclera strip increased. In (d) the strip is almost broken.

Scleral strips with a width of 2 mm were clamped horizontally with a distance of 5 mm between the jaws of the microcomputer-controlled biomaterial tester (Shanghai University; Shanghai; China). The sclera posterior to the limbus was secured in the jaw. Strain was increased linearly with a velocity of 1 mm/min and stress was measured up until tissue rupture (Figure 2). The strain-stress curve was recorded and the parameters stress (MPa) and Young's elastic modulus (MPa) from 2% to 14% of the strain of the samples were used for analysis [24].

### 2.5. Histology

All the eyes of FDMS and FDMG group perform histological examination. After the scleral strips used for biomechanical test were removed carefully, the left eye cups were fixed in 10% neutral buffered formalin for at least 24 hours for light microscopy. The specimens were embedded with paraffin. 4 mm thin sections were cut within and adjacent to the treatment area and stained with hematoxylin-eosin. The slides were examined using a light microscope (Leica DM4000B; Germany) at 40–1000 magnification.

### 2.6. Statistical Analysis

All the statistical analyses were processed using SPSS Version 11.5 software. The refraction and axial length of the right eye were statistically compared to the left eye within the same group prior to the experiment using the paired sample *t*-test. The biometric and stress-strain results for the right eye were compared between the different groups using one-way analysis of variance (ANOVA) with Bonferroni correction.

## 3. Results

Prior to the form deprivation (0 time-point; Table 1), there was no significant difference between the right eye and the left eye of the animals regarding refraction and axial length within each individual group (*p* > 0.05; paired sample *t*-test). The difference in refraction and axial length of the right eyes was not significant among all the four groups (refraction, *p* = 0.685; axial length, *p* = 0.949; one-way ANOVA with Bonferroni correction).

All of the right eyes of animals in the FDMG, FDM, and FDMS groups developed significant myopia in 4 weeks. Similar to the FDM group, the biometric parameters of the right eyes of animals in the FDMG and FDMS groups kept developing towards myopia during the 4-week observation period. Data regarding the development of refraction and axial length in the four groups is detailed in Table 1. At all time points the development of refraction and axial length in the deprived eyes of animals in the FDM, FDMG, and FDMS groups was similar but was significantly faster than the normal control group; the results were presented in Tables 1 and 2.

**Figure 3:**
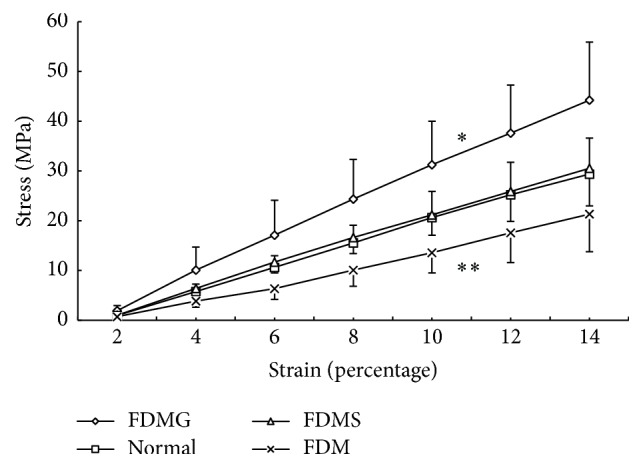
Stress-strain curve of the right eyes of all the four groups. A single asterisk indicates that the stress in the FDMG group was significantly higher than in the other groups (*p* < 0.05). A double asterisk indicates that the stress in the FDM group was lower than in the normal control group, when the strain was ≥6%; the difference was significant (*p* < 0.05). The stress in the FDMS group was similar to that in the normal control group.

**Figure 4:**
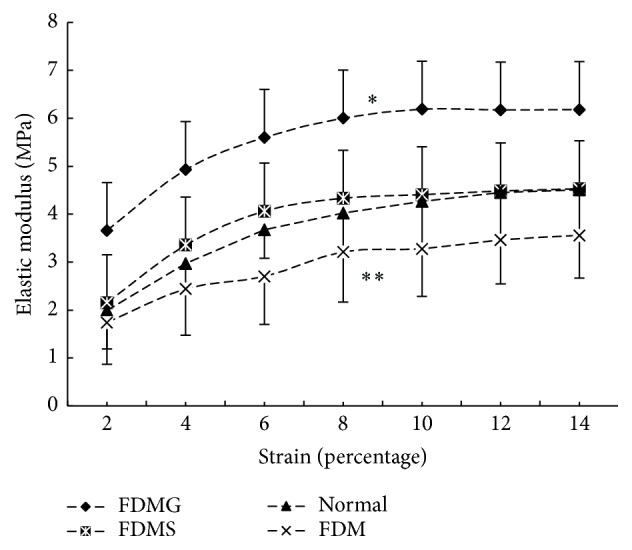
Differences in elastic modulus of the right eyes of all four groups. A single asterisk indicates that the elastic modulus in the FDMG group was significantly higher than in the other groups (*p* < 0.05). A double asterisk indicates that the elastic modulus in the FDM group was lower than in the normal control group; the difference was not significant (*p* > 0.05).

When compared with the normal control eyes, statistically significant changes occurred in the biomechanical parameters of the deprived eyes of animals in the FDMG, FDM, and FDMS groups (Table 3). The stress-strain curves and Young's elastic modulus-strain curves for all of the four groups are presented in Figures 3 and 4, respectively. For each group the stress-strain curve approximated to a straight line; however, when the thickness of the sclera was considered, there was not a single Young's elastic modulus at different strain levels. The elastic modulus increased from 2% strain and reached a value that was almost stable at 6% strain; the difference between the groups also reached almost stable value at 6% strain. The elasticity of the sclera in the eyes of animals in the FDM group increased; the stress in the deprived eyes of animals in the FDM group was significantly lower than normal control eyes, with the exception of the 2% and 4% strain levels. In contrast with the stress results, although Young's elastic modulus for the deprived eyes of animals in the FDM group was lower than in the normal control eyes, the difference was not significant at all strain levels. The results were presented in Table 3. Saline injection could improve the biomechanical properties of the sclera in the form-deprived eyes. The stress and Young's elastic modulus for the right eyes of animals in the FDMS group were both significantly higher than in the FDM group, with the exception of 2% and 4% strain (stress: FDMS versus FDM, 4% strain, *p* = 0.052, 6% strain *p* = 0.01, and 8% strain, *p* = 0.009; Young's elastic modulus: FDMS versus FDM, 4% strain, *p* = 0.072, 6% strain, *p* = 0.014, and 8% strain, *p* = 0.042; one-way ANOVA with Bonferroni correction). However, when compared to normal control eyes, there were no significant differences between the groups at all strain levels (Table 3).

Glyceraldehyde treatment also significantly enhanced the stiffness of the sclera. For the right eyes of animals in the FDMG group, the stress and Young's elastic modulus at all strain levels were significantly higher than for the right eyes of animals in all of the other groups (*p* < 0.01; one-way ANOVA with Bonferroni correction). The stress in the right eyes in animals in the FDMG group was 1.69 times greater and Young's elastic modulus was 1.08 times greater than in the right eyes of animals in the FDM group at 6% strain. The comparison of stress and Young's elastic modulus between FDMG and normal control group was showed in Table 3.

**Figure 5:**
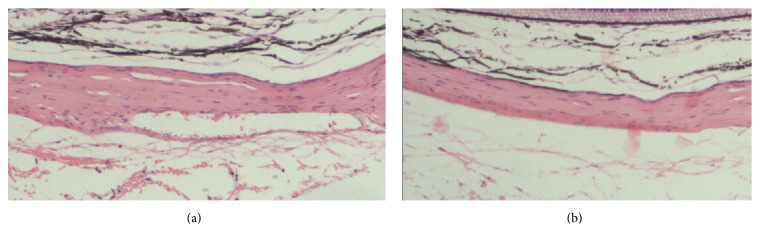
The Tenon capsule of the FDMG eye and the control eye. (a) The Tenon capsule in drug depot area of the FDMG eye became compact and adhered to the sclera tightly (H&E stain; original magnification -100). (b) The Tenon capsule was loose in the control eye (H&E stain; original magnification -100).

**Figure 6:**
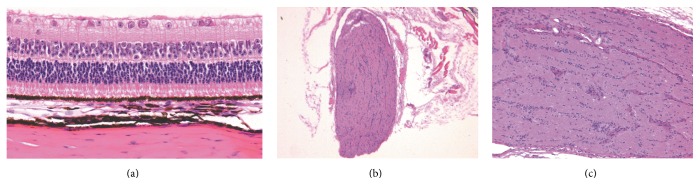
Intact retina and optic nerve in a glyceraldehydes treated eye. (a) The retina was without abnormalities (H&E stain; original magnification -400). ((b) and (c)) The optic never was intact (H&E stain; (b) original magnification -40; (c) original magnification -100).

Light microscopy examination showed that, in 7 eyes of FDMG group and 6 eyes in FDMS group, mild inflammatory infiltration or hemorrhage was seen in the episcleral tissue in the injection area. The Tenon capsule of the FDMG eyes became compact and regularly arranged. It adhered to the sclera tightly, but no scar has been observed (Figure 5). There was no inflammation in sclera. The optic nerve and retina in all samples were without abnormalities (Figure 6).

## 4. Discussion

In the present study, it was confirmed that form deprivation can cause significant myopia; in the eyes of guinea pigs with form-deprived myopia the sclera had weakened biomechanical properties. The stress in the eyes of animals in the FDM group was lower than that in the eyes of animals in the normal control group at all strain levels, and the difference was significant at 6% strain. Young's elastic modulus for the sclera was lower in the eyes of animals in the FDM group than in the normal control group, but the difference was not significant. These results were similar to those reported by Phillips and McBrien. In their study, the sclera extended over a distance that was 25% greater than in the controls at a load corresponding to 20 mmHg intraocular pressure; however, when the thickness of the sclera was taken into consideration, Young's elastic modulus was similar between the myopic and normal eyes under physiological pressure. Consequently, it was concluded that the alterations in elasticity were mainly the result of a thinner sclera in the eyes with myopia [25]. It is believed that scleral thinning in myopia is not the result of passive stretch of the sclera but is caused by active tissue remodeling. Decrease in the amount of collagen present and fibril diameter are two critical events that lead to scleral thinning and weakening. In an eye with a weakened sclera, physiological intraocular pressures may be sufficient to induce progressive ocular enlargement given sufficient time [2]. Consequently, treatment targeting scleral collagen may be effective in retarding the progression of myopia.

Experiments both* in vivo* and* in vitro* have demonstrated that the biomechanical parameters of sclera in normal eyes could be improved by treatment with glyceraldehyde [13, 16, 18]. Wollensak and Iomdina have demonstrated* in vivo* in rabbit sclera after glyceraldehyde treatment for 14 days that the ultimate stress increased by 409.7% and Young's modulus increased by 1027%; the ultimate strain decreased by 48.2% [18].* In vitro* after treatment with glyceraldehyde, the stress of treated porcine and human sclera increased by 487% and 34%, respectively [13]. In our study, it was found that in form-deprived eyes the biomechanical rigidity of the sclera could also be increased by treatment with glyceraldehyde. The stress and elastic modulus in the eyes of animals treated with glyceraldehydes was significant higher than in the eyes of animals in the normal control and FDM groups at all strain levels. This indicates that injected glyceraldehydes could overwhelm the scleral biomechanical changes caused by form deprivation. Through the Maillard reaction cascade, glyceraldehyde can be added to the ends of protein molecules and can be further transformed to more stable molecules called advanced glycation end products; this results in covalent collagen cross-links that can promote increased tissue stiffness and resistance to enzymatic degradation [26–28]. It had been demonstrated by ultrasound sub-Tenon injections can reach posterior sclera immediately and then disperse into surrounding tissue [29]. In this study repeated injections in four quadrants, respectively, facilitated glyceraldehydes reaching the whole posterior sclera which play most important role in the axial elongation.

Light microscopy examination showed the safety of glyceraldehyde sub-Tenon injection. The mild inflammation possibly related to injection. Abnormalities of the retina and optic never have not been observed in our study; the same results have also been reported by Wollensak and Iomdina [18]. The Tenon capsule of the FDMG eyes became compact and adhered to the sclera tightly that may be caused by injections and glyceraldehydes. The result of these changes has not been studied furtherly, but there was no scar observed and the injections have been done well in this study.

In the current study, although the injection of glyceraldehydes increased scleral rigidity, the development of myopia was not retarded in experimental eyes relative to the normal control eyes. All of the biometric parameters regarding the eyes of animals in the FDMG group were not significantly different from those in the FDM group at all time points. The results of other studies aimed at evaluating the efficiency of sclera reinforcement have been controversial. Scleral strengthening by means of polymer injection to slow ocular elongation has been studied in animal models and humans; the results were reciprocal. Su et al. reported that polymeric hydrogels, either implanted or injected adjacent to the outer scleral surface, could not slow ocular elongation in chicken eyes, although the sclera thickness was significantly increased [30]. The authors considered that a possible reason for this result relates to the bilayered structure of the chick sclera; the stiffer cartilage component of the chick sclera may determine the rate of elongation. In mammalian and primate eyes that have monolayered fibrous scleras, the addition of the fibrous capsule is likely to have a greater impact on scleral biomechanical properties and could possibly slow ocular relongation [30]. Indeed, it has been reported by Avetisov et al. that repeated injections of liquid polymeric composition could promote collagen formation in 146 rabbit eyes and slow myopia progression in 240 human eyes [6]. This finding is at odds with our observations regarding form-deprived myopia in the guinea pig. The axial elongation could not have been retarded by increased scleral stiffness in our study. One possible reason may be that myopia was progressive and more pronounced in the former study. The reduction in scleral thickness is progressive during the development of myopia [31]. In addition to this, a reduction in collagen fibril diameter has been reported to occur at the posterior pole [32]. In experimental myopia, the collagen fibril diameter has been found to decrease only after a long period of myopia (3 months in the tree shrew) [33]. Therefore, collagen change may not be the most important factor that contributes to the progression of short-term experimental myopia. The improvement in scleral rigidity based on collagen cross-linking may be more effective in long-term progressive myopia. On the other hand, the results of our study may indicate that enhancement of scleral biomechanical properties does not alone guarantee slowed eye growth in the FDM guinea pig eye model, consistent with suggestions by McBrien and Norton. They found that form-deprived myopia could be increased by administration of aminopropionitrile, which could prevent collagen cross-linking; at the same time it was also found that eyes treated with aminopropionitrile without form deprivation were no different from normal untreated eyes. It appears that focused images falling on the retina could control ocular development in a growing eye despite the presence of structural changes involving collagen. Several factors are involved in the progression of myopia, a predisposition to myopia or other error signals combined with abnormal collagen structure, and work together to produce myopia [10]. It is reasonable to assume that if the reinforcement of sclera reaches a sufficiently high level it can retard myopia by overwhelming all of the factors leading to its progression. But to make the sclera more rigidity may be combined with more side effects.

In summary, the results of the present study represent proof that the scleral biomechanical properties of form-deprived eyes can be improved using sub-Tenon injection of glyceraldehydes; however, the rate of ocular elongation was not affected. Because of differences in high and moderate myopia, a follow-up study involving progressive high and long-term myopia in a mammalian animal models will be necessary to establish their suitability for myopia control with collagen cross-linking.

## Figures and Tables

**Table 1:** Biometric results of all the four groups (mean ± SD) and the comparison of refraction and axial length between FDM, FDMS, FDMG, and normal control group, respectively (one-way ANOVA with Bonferroni correction).

Time point (week)	Refraction (diopter)		*p* ^*∗*^ (the right eye)	Axial length (mm)		*p* ^*∗∗*^ (the right eye)
	Right	Left		Right	Left	
Normal						
0	3.39 ± 1.54	3.28 ± 1.39		6.97 ± 0.11	6.97 ± 0.09	
2	1.53 ± 1.2	1.42 ± 1.29		7.32 ± 0.09	7.47 ± 0.06	
4	0.61 ± 0.93	0.22 ± 0.75		7.50 ± 0.11	7.61 ± 0.12	

FDM						
0	3.28 ± 1.26	3.19 ± 1.98		6.97 ± 0.17	6.89 ± 0.20	
2	0.11 ± 0.8	1.36 ± 1.42	0.001	7.51 ± 0.12	7.45 ± 0.12	0.028
4	−1.28 ± 0.68	0.06 ± 0.94	0.000	7.72 ± 0.13	7.62 ± 0.07	0.008

FDMS						
0	2.89 ± 1.53	3 ± 2.32		7.03 ± 0.25	6.97 ± 0.19	
2	−0.25 ± 1.03	1.03 ± 1.56	0.009	7.55 ± 0.17	7.45 ± 0.10	0.005
4	−1.39 ± 0.63	−0.19 ± 1.34	0.000	7.76 ± 0.15	7.68 ± 0.13	0.001

FDMG						
0	2.72 ± 1.34	2.92 ± 1.48		6.97 ± 0.37	6.92 ± 0.49	
2	−0.39 ± 1.21	0.97 ± 0.96	0.004	7.56 ± 0.13	7.50 ± 0.18	0.003
4	−1.47 ± 1.34	−0.25 ± 1.33	0.001	7.84 ± 0.15	7.73 ± 0.14	0.000

“*p* ^*∗*^” refers to the difference of refraction between the FDM, FDMS, FDMG, and normal control group, respectively.

“*p* ^*∗∗*^” refers to the difference of axial length between the FDM, FDMS, FDMG, and normal control group, respectively.

**Table 2:** The comparison of refraction and axial length of the right eyes among FDM, FDMS, and FDMG group at all time point (one-way ANOVA with Bonferroni correction).

Group	Refraction (diopter)			Axial length (mm)		
	0 week	2 weeks	4 weeks	0 week	2 weeks	4 weeks
FDM	3.28 ± 1.26	0.11 ± 0.8	−1.28 ± 0.68	6.97 ± 0.17	7.51 ± 0.12	7.72 ± 0.13
FDMS	2.89 ± 1.53	−0.25 ± 1.03	−1.39 ± 0.63	7.03 ± 0.25	7.55 ± 0.17	7.76 ± 0.15
FDMG	2.72 ± 1.34	−0.39 ± 1.21	−1.47 ± 1.34	6.97 ± 0.37	7.56 ± 0.13	7.84 ± 0.15
*p*	0.685	0.578	0.908	0.949	0.701	0.194

**Table 3:** The comparison of stress and Young's elastic modulus between FDM, FDMS, FDMG, and normal control group, respectively (one-way ANOVA with Bonferroni correction).

Group	Strain (percentage)	Stress (Mpa)	*p* ^*∗*^	Young's elastic modulus (Mpa)	*p* ^*∗∗*^
Normal	2	0.97 ± 0.43		1.99 ± 0.8	
	6	10.65 ± 2.33		3.67 ± 0.59	
	10	20.62 ± 5.28		4.27 ± 0.88	
	14	29.38 ± 7.23		4.51 ± 0.89	

FDM	2	0.74 ± 0.41	0.456	1.74 ± 0.87	0.291
	6	6.37 ± 2.83	0.034	2.70 ± 1.0	0.074
	10	13.60 ± 5.12	0.028	3.28 ± 1.0	0.067
	14	21.33 ± 6.72	0.016	3.56 ± 0.90	0.109

FDMS	2	1.02 ± 0.57	0.881	2.15 ± 1.28	0.436
	6	11.69 ± 2.16	0.595	4.07 ± 0.88	0.464
	10	21.17 ± 4.07	0.647	4.41 ± 0.91	0.789
	14	30.54 ± 7.56	0.768	4.53 ± 1.11	0.807

FDMG	2	1.92 ± 1.03	0.005	3.66 ± 1.25	0.000
	6	17.12 ± 7.0	0.002	5.60 ± 1.70	0.001
	10	31.28 ± 8.72	0.001	6.19 ± 1.53	0.001
	14	44.22 ± 11.69	0.001	6.18 ± 1.65	0.002

“*p* ^*∗*^” refers to the difference of stress between the FDM, FDMS, FDMG, and normal control group, respectively. “*p* ^*∗∗*^” refers to the difference of Young's elastic modulus between the FDM, FDMS, FDMG, and normal control group, respectively.
